# Primary and secondary postpartum haemorrhage: a review for a rationale endovascular approach

**DOI:** 10.1186/s42155-024-00429-7

**Published:** 2024-02-13

**Authors:** Alberto Alonso-Burgos, Ignacio Díaz-Lorenzo, Laura Muñoz-Saá, Guillermo Gallardo, Teresa Castellanos, Regina Cardenas, Luis Chiva de Agustín

**Affiliations:** 1 Radiology Department, Vascular Surgery and Interventional Radiology Unit, University Clinic of Navarra, Clínica Universidad de Navarra, Marquesado de Santa Marta 1, 28027 Madrid, Spain; 2 Radiology Department, Interventional Radiology Unit, University Hospital La Princesa, Madrid, Spain; 3 Radiology Department, University Clinic of Navarra, Madrid, Spain; 4 Gynaecology and Obstetrics, University Clinic of Navarra, Madrid, Spain

**Keywords:** Postpartum haemorrhage, Uterus atony, Retained products conception, Placenta acreta, Embolization

## Abstract

Postpartum haemorrhage (PPH) is a significant cause of maternal mortality globally, necessitating prompt and efficient management. This review provides a comprehensive exploration of endovascular treatment dimensions for both primary and secondary PPH, with a focus on uterine atony, trauma, placenta accreta spectrum (PAS), and retained products of conception (RPOC). Primary PPH, occurring within 24 h, often results from uterine atony in 70% of causes, but also from trauma, or PAS. Uterine atony involves inadequate myometrial contraction, addressed through uterine massage, oxytocin, and, if needed, mechanical modalities like balloon tamponade. Trauma-related PPH may stem from perineal injuries or pseudoaneurysm rupture, while PAS involves abnormal placental adherence. PAS demands early detection due to associated life-threatening bleeding during delivery. Secondary PPH, occurring within 24 h to 6 weeks postpartum, frequently arises from RPOC. Medical management may include uterine contraction drugs and hemostatic agents, but invasive procedures like dilation and curettage (D&C) or hysteroscopic resection may be required.

Imaging assessments, particularly through ultrasound (US), play a crucial role in the diagnosis and treatment planning of postpartum haemorrhage (PPH), except for uterine atony, where imaging techniques prove to be of limited utility in its management. Computed tomography play an important role in evaluation of trauma related PPH cases and MRI is essential in diagnosing and treatment planning of PAS and RPOC.

Uterine artery embolization (UAE) has become a standard intervention for refractory PPH, offering a rapid, effective, and safe alternative to surgery with a success rate exceeding 85% (Rand T. et al. CVIR Endovasc 3:1-12, 2020). The technical approach involves non-selective uterine artery embolization with resorbable gelatine sponge (GS) in semi-liquid or torpedo presentation as the most extended embolic or calibrated microspheres. Selective embolization is warranted in cases with identifiable bleeding points or RPOC with AVM-like angiographic patterns and liquid embolics could be a good option in this scenario. UAE in PAS requires a tailored approach, considering the degree of placental invasion. A thorough understanding of female pelvis vascular anatomy and collateral pathways is essential for accurate and safe UAE.

In conclusion, integrating interventional radiology techniques into clinical guidelines for primary and secondary PPH management and co-working during labour is crucial.

**Graphical Abstract:**

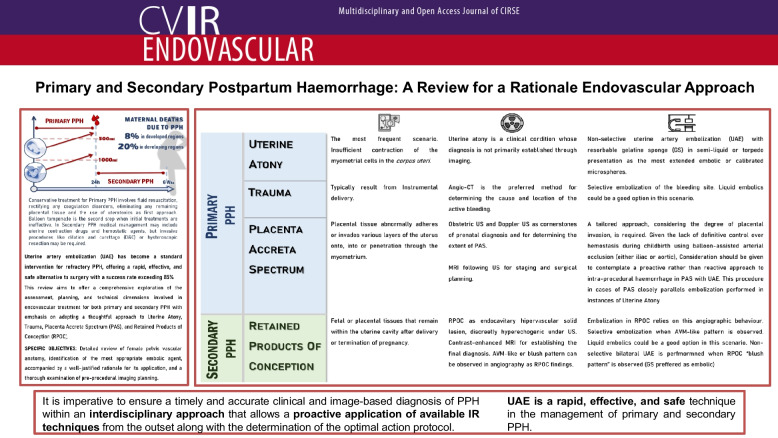

## Background

**Fig. 1:**
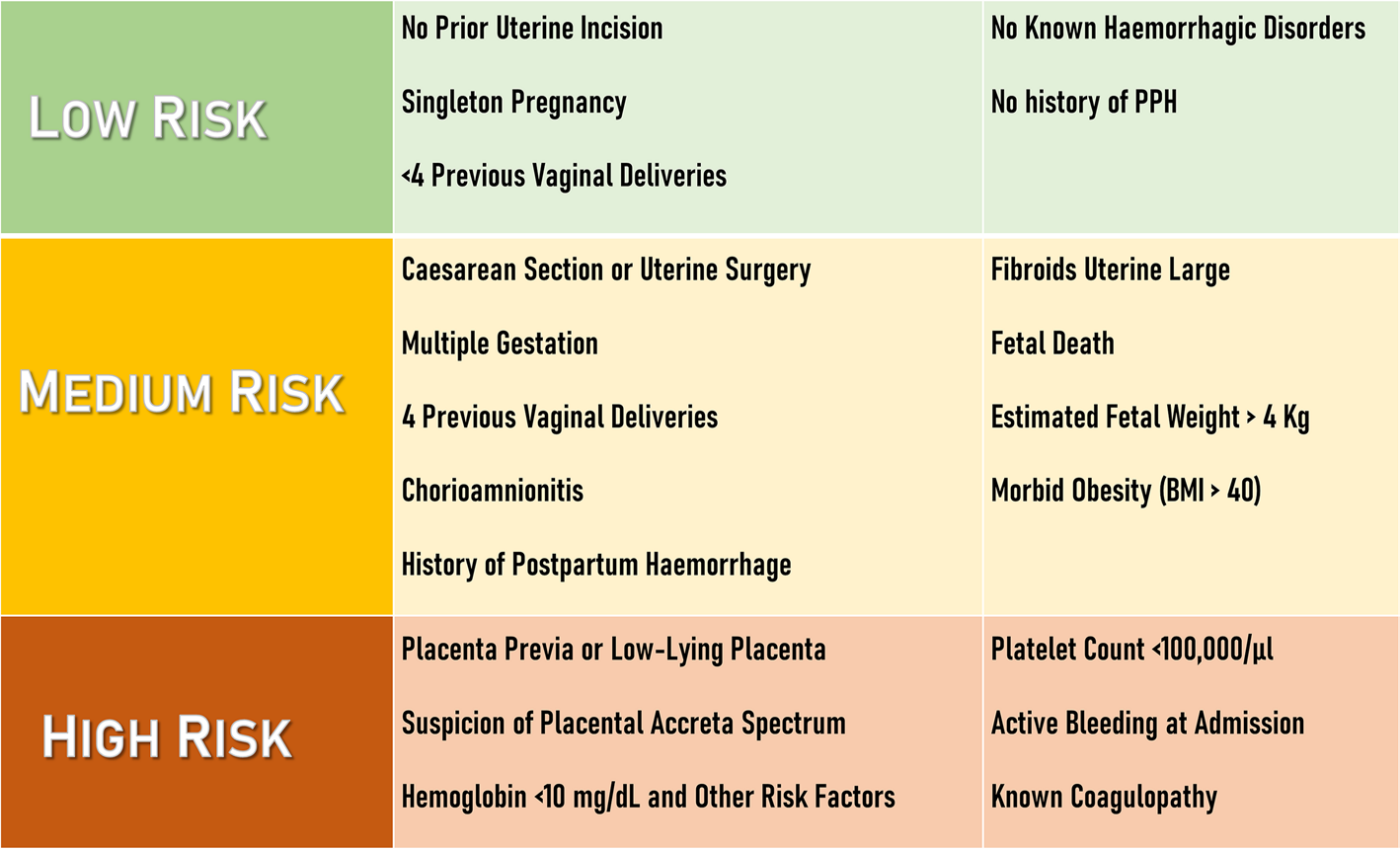
Resume of predictive risk factors associated to PPH

Postpartum haemorrhage (PPH) is defined as blood loss of over 500 ml following a vaginal delivery or over 1000 ml after a cesarean section delivery [1]. PPH represents 8% of maternal deaths in developed regions and 20% of maternal deaths in developing regions [1, 2]. The assessment of PPH risk factors (summarized in Fig. 1) can prove beneficial in preparing for delivery [1, 3].

In the face of PPH, an immediate and sequential intervention is paramount, with the initial approach centred on maintaining and/or restoring the haemodynamic stability of the patient. An obstacle in efficient management of PPH is its frequent late detection, but even more crucial is the decision-making to recognize the condition and implement corrective measures [1, 4]. The initial approach to PPH involves fluid resuscitation, rectifying any coagulation disorders, eliminating any remaining placental tissue and the use of uterotonics [1–5]. Balloon tamponade (i.e. Bakri® balloon) is the secondary treatment for PPH when initial treatments are ineffective. This strategy effectively halts bleeding in 85% of cases [3, 6].

This review aims to offer a comprehensive exploration of the assessment, planning, and technical dimensions involved in endovascular treatment for both primary and secondary PPH with emphasis on adopting a thoughtful approach to UA, trauma, placenta accreta spectrum, and retained products of conception. The specific objectives encompass a detailed review of female pelvis vascular anatomy, identification of the most appropriate embolic agent, accompanied by a well-justified rationale for its application, and a thorough examination of pre-procedural imaging planning.

## Primary and secondary postpartum hemorraghe

Primary PPH refers to PPH occurring within the initial 24 h. Secondary PPH is defined as significant vaginal bleeding that occurs between 24 h following placental delivery and throughout the subsequent 6 weeks [1, 4]. The main causes for both primary and secondary PPH are summarized in Table 1 [1, 5].

**Table 1:** The four T mnemonic. Causes, incidences and factors associated with PPH

	**CAUSES**	**RELATED FACTORS**
**UTERINE ATONY (TONE, 70% incidence)**	**Uterine overdistension**	Multiple gestation Hydramnios Macrosomic fetus
	**Chorioamnionitis**	Prolonged rupture of membranes Fever
	**Muscle exhaustion**	Prolonged and/or rapid labour High multiparity
**TISSUE RETENTION (TISSUE, 10% incidence)**	**Placenta**	Placenta accreta Previous uterine surgery
	**Clots**	
**BIRTH CANAL INJURY (TRAUMA, 20% incidence)**	**Tears in the birth canal**	Instrumental delivery Precipitous second stage
	**Uterine rupture or dehiscence**	Previous uterine surgery (cesarean section) Instrumental delivery Dystocia Hyperactivity External cephalic version
	**Uterine inversion**	Manual removal of the placenta Placenta accreta Credé maneuver
**COAGULATION DISORDERS (THROMBIN, 1% incidence)**	**Acquired**	Preeclampsia HELLP syndrome Disseminated intravascular coagulation (DIC) Amniotic fluid embolism Sepsis Placental abruption
	**Congenital**	Von Willebrand disease Hemophilia type A

### Primary postpartum haemorrage

#### Uterus atony

Uterus Atony (UA) refers to the insufficient contraction of the myometrial cells in the corpus uteri in response to the release of endogenous oxytocin. If the uterus does not contract adequately, spiral arteries might keep bleeding, leading to PPH.

The bimanual uterine massage assumes a pivotal role as the primary measure to induce uterine contractions by instigating the release of endogenous prostaglandins. Concurrently, oxytocin, administered intravenously or intramuscularly, emerges as the principal therapeutic intervention for the management of PPH attributed to UA. In instances where pharmacotherapy proves inadequate, supplementary agents like methylergonovine maleate and intramuscular prostaglandins such as carboprost tromethamine can be deployed. In the event of pharmacotherapeutic limitations, the implementation of mechanical modalities becomes imperative. Balloon tamponade, as exemplified by the Bakri balloon, stands as a noteworthy technique in this regard [1].

#### Trauma

**Fig. 2:**
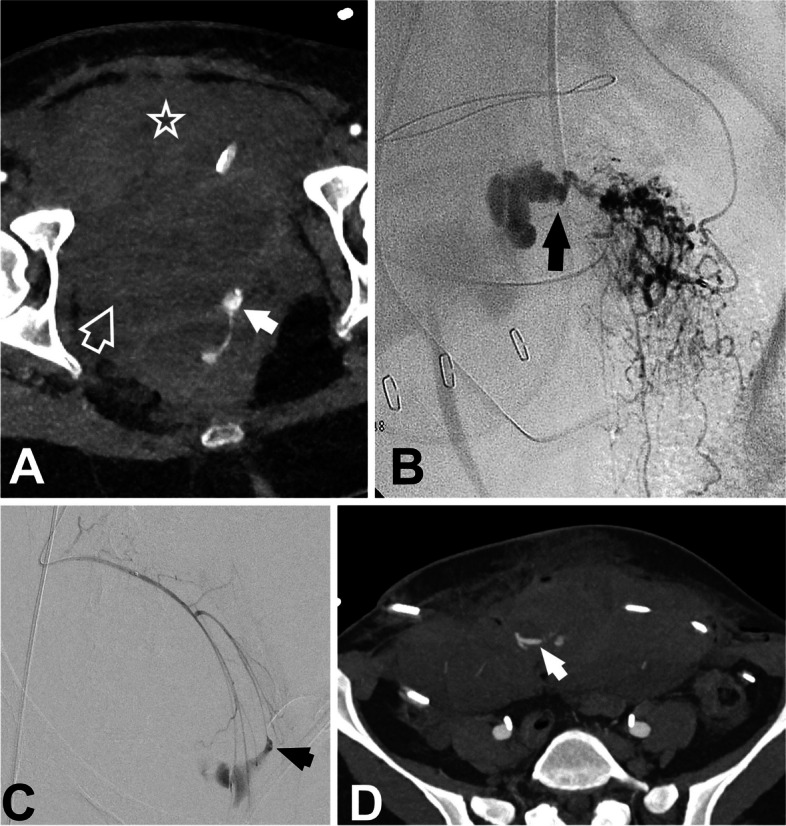
**A** 36-year-old postpartum patient with hemodynamic instability and a sudden drop in hematocrit in the immediate postpartum period following an instrumental delivery with spatulas, despite the absence of significant vaginal bleeding. Angio-CT with arterial and delayed contrast phase was performed, revealing active extrauterine bleeding at the cervicovaginal level (arrow) with the formation of hematomas in the perivesical (open arrow) and prevesical (hollow star) spaces. **B** Selective catheterization and angiography in uterine pseudoaneurism rupture after cesarean delivery with active bleeding (arrow). **C** Selective catheterization and angiography in cervical pseudoaneurism rupture after cesarean delivery with active bleeding (arrow) after forceps-assisted instrumental delivery. **D** 32-year-old patient in immediate postpartum period after cesarean delivery. A significant increase in abdominal circumference is observed, along with a decrease in hematocrit and signs of hemodynamic instability. Hematoma of the rectus sheath with signs of active bleeding (arrow) was observed in angioCT

More than 85% of women who undergo vaginal delivery are highly likely to encounter some degree of perineal trauma, with up to 70% of these cases necessitating sutures for proper repair [7]. The most severe lesions typically result from the use of instruments. Large cervical tears (> 2 cm) or those accompanied by significant bleeding should be promptly addressed [7], as they progress, these hematomas may extend into the preperitoneal, prevesical, and perivesical spaces (Fig. 2A).

The rupture of a pseudoaneurysm in the uterine or vaginal artery is a rare cause of PPH (Fig. 2B, C), occurring in approximately 3% of cases, with most cases being associated to cesarean deliveries [8].

The rectus sheath is a common site for extrauterine PPH as a result from damage to the deep inferior epigastric arteries in cesarean (Fig. 2D) delivery but also due to disseminated intravascular coagulation (DIC) or inadequate hemostasis [9].

#### Placenta accreta spectrum

The Placenta Accreta Spectrum (PAS) refers to a condition in which placental tissue abnormally adheres or invades various layers of the uterus onto the myometrium (placenta accreta, FIGO grade 1), infiltration into the myometrium (placenta increta, FIGO grade 2), or even penetration through the myometrium to surrounding organs (placenta percreta, FIGO grade 3) [10, 11]. The incidence of PAS run in parallel with the escalating rate of cesarean section procedures, and present incidence is one in every 500 births [10, 11]. PAS represents one of the gravest complications during pregnancy, turning the pelvis into a highly vascular state and in risk of life-threatening bleeding throughout the pregnancy, but it reaches a critical level during delivery. This risk of bleeding increases as the degree of placental invasion rises [11]. In this sense, Cali et al. [12] highlighted the depth of placental invasion as a pivotal outcome factor, where less invasive PAS exhibited reduced bleeding. Nevertheless, to this day, up to 50% of PAS cases remain undiagnosed until delivery [10]. Women with risk factors for PAS, such as a history of placenta previa or cesarean section, should have their ongoing pregnancies evaluated and monitored at centres with expertise in this condition [10, 11].

The primary treatment for PAS involves a cesarean hysterectomy, with the placenta left in place after the delivery. This treatment often leads to complications, such as prolonged surgical procedures, lower urinary tract injuries and intensive care unit admissions [11]. Up to 95% of women affected by placenta accreta require blood transfusions, with approximately one-third of these involving 10 or more units of red blood cells within 24 h (massive transfusions) [11, 13]. A team-based, patient-centric, and evidence-based approach is essential from diagnosis through to complete recovery [13].

### Secondary postpartum haemorrage

#### Retained products of conception

Retained Products of Conception (RPOC) refers to fetal or placental tissues that remain within the uterine cavity after delivery or termination of pregnancy. RPOC may lead to the persistence and even expansion of physiological maternal arteriovenous shunting in the placental giving a marked vascularity or even an arteriovenous malformation (AVM) behaviour [14–16]. This hypervascular nature of the lesion underlies to being one of the leading causes of secondary PPH [1].

Medical management of RPOC, including uterine contraction drugs (oxytocin, misoprostol or methylergometrine) and hemostatic agent (carbazo-chrome and tranexamic acid) is not always effective. Invasive therapies involve the removal of the retained tissue through dilation and curettage (D&C) or hysteroscopic resection [15, 17–19]. A precise imaging-based diagnostic approach and a tailored and multidisciplinary approach will aid in identifying patients for whom expectant management is appropriate and those requiring a more aggressive intervention [15, 18, 19].

### Imaging assesment in pph

#### Uterus atony

Uterine atony is a clinical condition whose diagnosis is not primarily established through imaging: US or Doppler US plays a limited role in the context of acute PPH resulting from UA, and likewise, CT scanning is not the recommended initial diagnostic approach. Recurrent bleeding following primary PPH endovascular treatment occurs in 5–10% of patients due to arterial spasms, or collateral vessels [20]. In this sense, angio-CT may prove beneficial to stablish the exact bleeding site.

#### Trauma

US can identify pelvic extraperitoneal hematomas, nevertheless, angio-CT is the preferred method for determining the cause and location of the active bleeding, observed as the extravasation of intravenous contrast material (Fig. 2A) [9]. In our institution, we conduct a standardized two-phase contrast-enhanced CT scan, consisting of an arterial phase and a delayed phase, to assess intra-abdominal bleeding, including trauma-related PPH. CT scan is performed using 100 mL of contrast media at a flow rate of 4 mL/s. Arterial phase is carried out using bolus tracking technique with region of interest (ROI) positioned at the L2 level, and a 15-s acquisition delay. The delayed phase scanning is consistently performed with a fixed delay of 60 s.

#### Placenta accreta spectrum

Accurate prenatal diagnosis of the degree of placental invasion are crucial for determining the most appropriate tailored procedure to each case and have demonstrated to improve outcomes in terms of maternal morbidity and mortality [11].

**Fig. 3:**
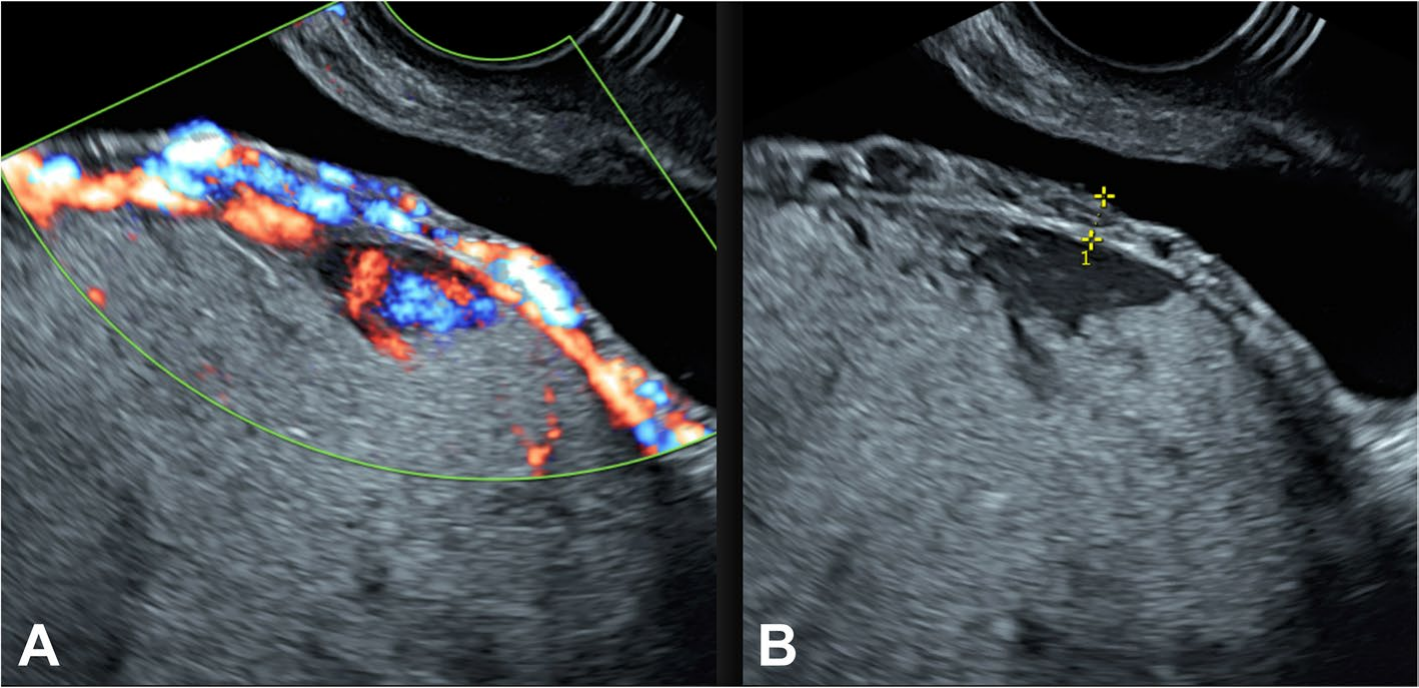
US and Doppler US study in PAS reveals the presence of abnormal placental lacunae, interruption of the bladder wall, myometrial thinning, and uterovesical hypervascularity

Obstetric US and Doppler US are the cornerstones of prenatal diagnosis for PAS, as well as for determining the extent of the lesion (Fig. 3), with sensitivity and specificity of 80 and 90% [11, 21]. Although MRI has not shown superiority to US in the diagnosis of PAS, patients should undergo MRI following US for staging and surgical planning [13]. This approach is particularly valuable in instances of posterior PAS or suspected bladder invasion [11, 22]. It is important to note, however, that both US and MRI studies in PAS are subject to variability in sensitivity and specificity, contingent upon working group's experience in this condition [10, 11]. Suboptimal imaging assessment, both in US and MRI, diagnosis may, in part, stem from terminology discrepancies to the same observed abnormality. PAS can be identified in US and MRI as summarized in Table 2 [21, 22].

**Table 2:** Standardized terms for US and MRI in PAS imaging assessment

US AND DOPPLER US	MRI
Loss of the 'clear zone' (absence or irregularity of the hypoechoic plane in the myometrium beneath the placental bed)	Heterogeneous Placenta
Abnormal placental lacunae	Placental Bulge
Bladder wall interruption	Dark Intraplacental Bands (hypointensity with a linear appearance, visible on T2W images)
Myometrial thinning (< 1 mm)	Placental Ischemic Infarction (areas of increased signal intensity on T2W images and decreased signal intensity on T1W images)
Placental bulge (deviation of the uterine serosa)	Loss of Retroplacental Dark Zone
Focal exophytic mass	Myometrial Thinning (< 1 mm observed on T2W images)
Uterovesical hypervascularity	Bladder Wall Interruption
Subplacental hypervascularity	Focal Exophytic Mass
Bridging vessels (traversing the myometrium and extending beyond the serosa)	Abnormal Vascularization of the Placental Bed
Placental lacunae feeder vessels	
Intraplacental hypervascularity	

#### Retained products of conception

**Fig. 4:**
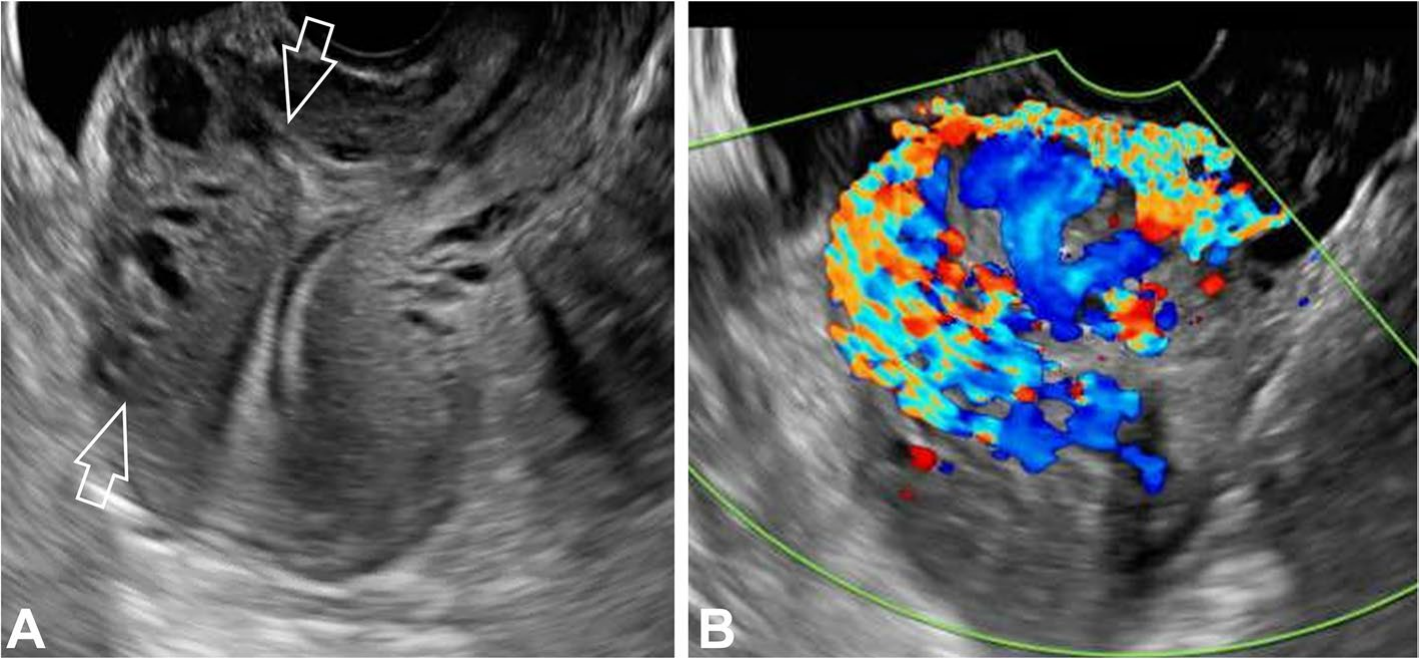
Transvaginal Doppler US of RPOC observed as a heterogeneous endocavitary mass (open arrows in A) with marked vascularity on Doppler-US (image B)

Under US, RPOC will be identified as an endocavitary solid lesion, discreetly hyperechogenic (Fig. 4). In Doppler-US, RPOC will exhibit marked hypervascularization and a high-flow, low-resistance waveform pattern behavior akin to that of an AVM [15, 23]. The presence of this highly vascular uterine lesion displaying AVM-like imaging behavior in the context of a woman presenting with secondary PPH should raise suspicion of RPOC as the primary diagnostic consideration since congenital uterine vascular malformations represent an exceptionally rare entity [15, 16, 18]. Being the thin endometrial stripe the most practically useful data in symptomatic patients, US criteria for embolization Vs. conservative treatment in RPOC is summarized in Table 3 [23, 24].

**Table 3:** US criteria suggested for conservative Vs. embolization treatment in RPOC

CONSERVATIVE MANAGEMENT	EMBOLIZATION
Endometrial Thickness < 10 mm	Endometrial Thickness > 10 mm
Lack of Hipervascular Lesion	Increased Myometrial Vascularity
Absence of Endometrial Vascularity	Vascularized Endometrium
	Resistance Indices < 0.48
	Peak Systolic Velocities > 0.82 m/s

**Fig. 5:**
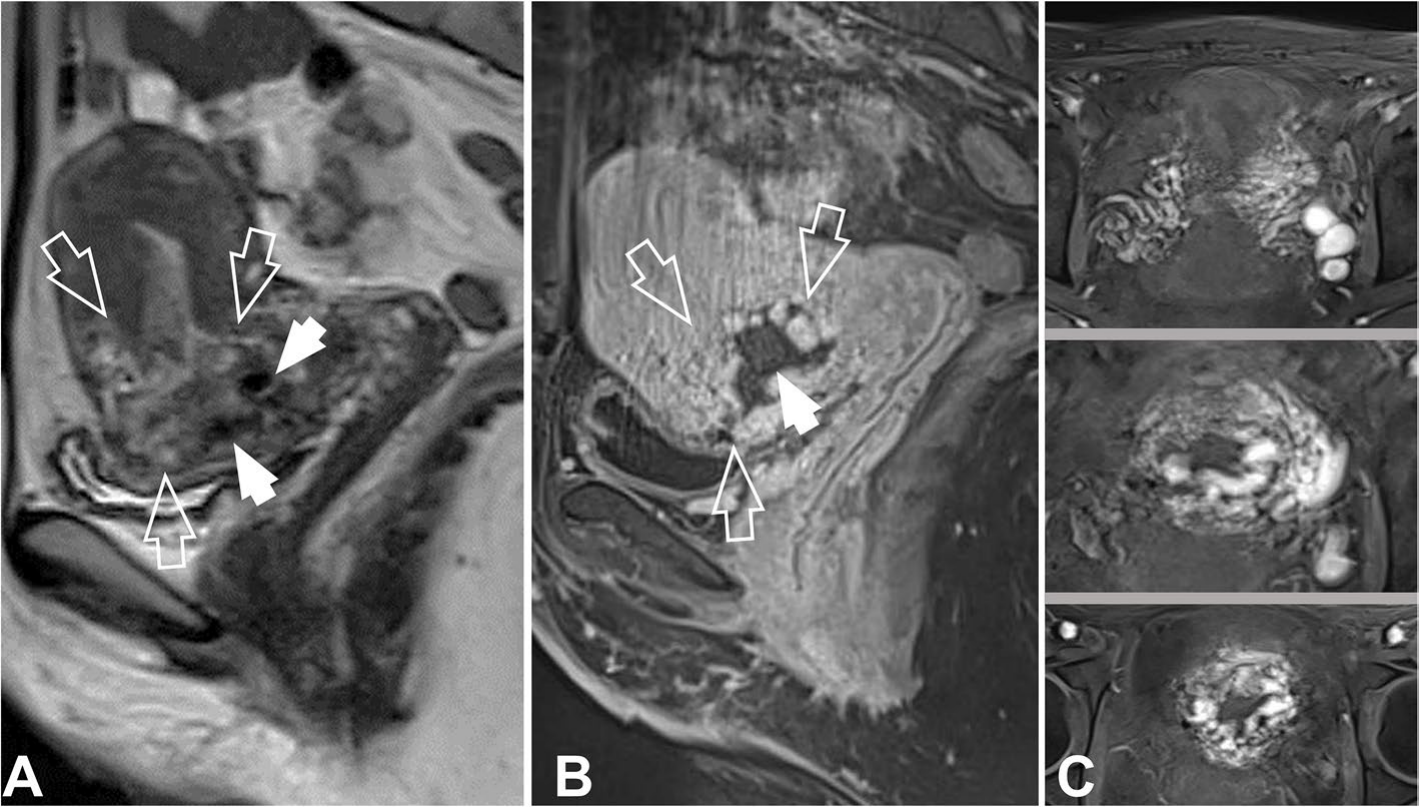
RPOC in a 38-year-old woman with mild secondary PPH (40 days after delivery), characterized as a heterogeneously intense rounded mass, was noted, manifesting a hyperintense signal in T2-weighted imaging and a corresponding hypointense signal in T1-weighted imaging (open arrows in A and B, respectively). Furthermore, multiple regions exhibiting signal void indicative of high-flow vascularization, were observed (arrows in A—B). Contrast-enhanced imaging revealed pronounced arterial phase vascular recruitment and early enhancement (image C)

Both CT and MRI are recommended techniques for the diagnostic assessment of patients with secondary PPH and when the presence of RPOC is suspected through US but MRI is likely the technique of choice, not only for establishing the final diagnosis of RPOC but also for assessing other less common primary uterine causes of secondary PPH [15]. Under contrast-enhanced MRI, RPOC will be identified as a rounded mass with areas of signal void corresponding to high-flow vascularization (Fig. 5). The early arterial phase study will reveal significant signal hyperintensity with marked enhancement associated with early venous drainage [18].

### Current role and principles for uterine artery embolization in PPH

Traditionally, PPH refractory to conservative treatment has required urgent surgical intervention, with a high rate of complications and a 4% mortality rate [1]. Presently, uterine artery embolization (UAE) has significantly reduced mortality and complications, resulting in a gradual decrease in the postpartum hysterectomy rate, from 1/1,000 to 1/2,000 over the past 20 years [3, 20].

UAE is a safer and effective alternative compared to conventional surgical approach for both Primary and Secondary PPH with a clinical success rate from 79 to 100% [20] but also considered as a standard approach for refractory PPH [3, 20]. This requires the availability of skilled interventional radiologists, adequate imaging facilities, and a collaborative interdisciplinary team [3, 20]. UAE has demonstrated its safety in PPH, with complications rates from 4 to 18% [20]. In skilled hands, incidents of non-target embolization are infrequent, and instances of uterine necrosis are due to excessive over-embolization, the use of inadequately small particles, presence of arterio-venous shunts or the disruption of collateral blood supply due to prior ligations [20].

Predictive factors associated with an unsuccessful UAE in PPH encompass DIC, blood transfusion of 5—10 units, blood loss > 1.5L, pronounced arterial vasoconstriction during embolization procedure and unilateral UAE [25].

According to Matsuzaki et al. and Radan et al., 91–100% of women who undergo UAE for primary or secondary PPH, experience a return to regular menstrual cycles without adverse effects on fertility and without causing harm to the endometrium [26, 27]. In most cases involving UAE for PPH, full-term pregnancies occur spontaneously, however, first trimester miscarriage rates are increased [27]. Similar pregnancy rates of approximately 60% have been documented in both women treated with temporary or permanent embolic agents [27, 28]. Furthermore, no statistically significant association has been identified between the embolic agent used and the abortion rate [27, 28].

### Indications, workflow and technical approach

The technical principles and workflow for UAE for PPH align with pelvic trauma scenarios; embolization should be rapid and efficient, aiming to prevent over-embolization and non-targeted embolization. In this sense, continuous maternal monitoring, haemodynamic support, and a dedicated anaesthetic are mandatory for PPH endovascular treatment procedure [1, 20].

Transfemoral approach (TFA) is the most employed in UAE [20], however the transradial approach (TRA) has been steadily gaining popularity. According to Himiniuc et al. and Khayrutdinov et al., TRA currently stands as a valuable technique when compared to TFA in UAE, due to its reduced risk of complications (3.7% for TFA Vs. 1.4% for TRA) [29, 30]. The average procedure time for UAE in PPH due to UA via TFA amounts to 90 min [31]. However, the use of the TRA in UAE yields similar effectiveness while reducing radiation exposure and procedure duration [30]. However, while TRA may be effective in elective situations, its utility might be less clear in emergency scenarios, particularly when peripheral issues and significant vascular spasm are present.

PPH embolization procedure involves initiating a pelvic angiogram followed by selective injections into the internal iliac artery to identifying the point of origin of the uterine artery. The uterine arteries can be easily catheterized with a 4Fr or 5-Fr Cobra or Multipurpose shape catheter, 125 cm or 150 cm length when performing a TRA and 65–80 cm when using a TFA.

**Fig. 6:**
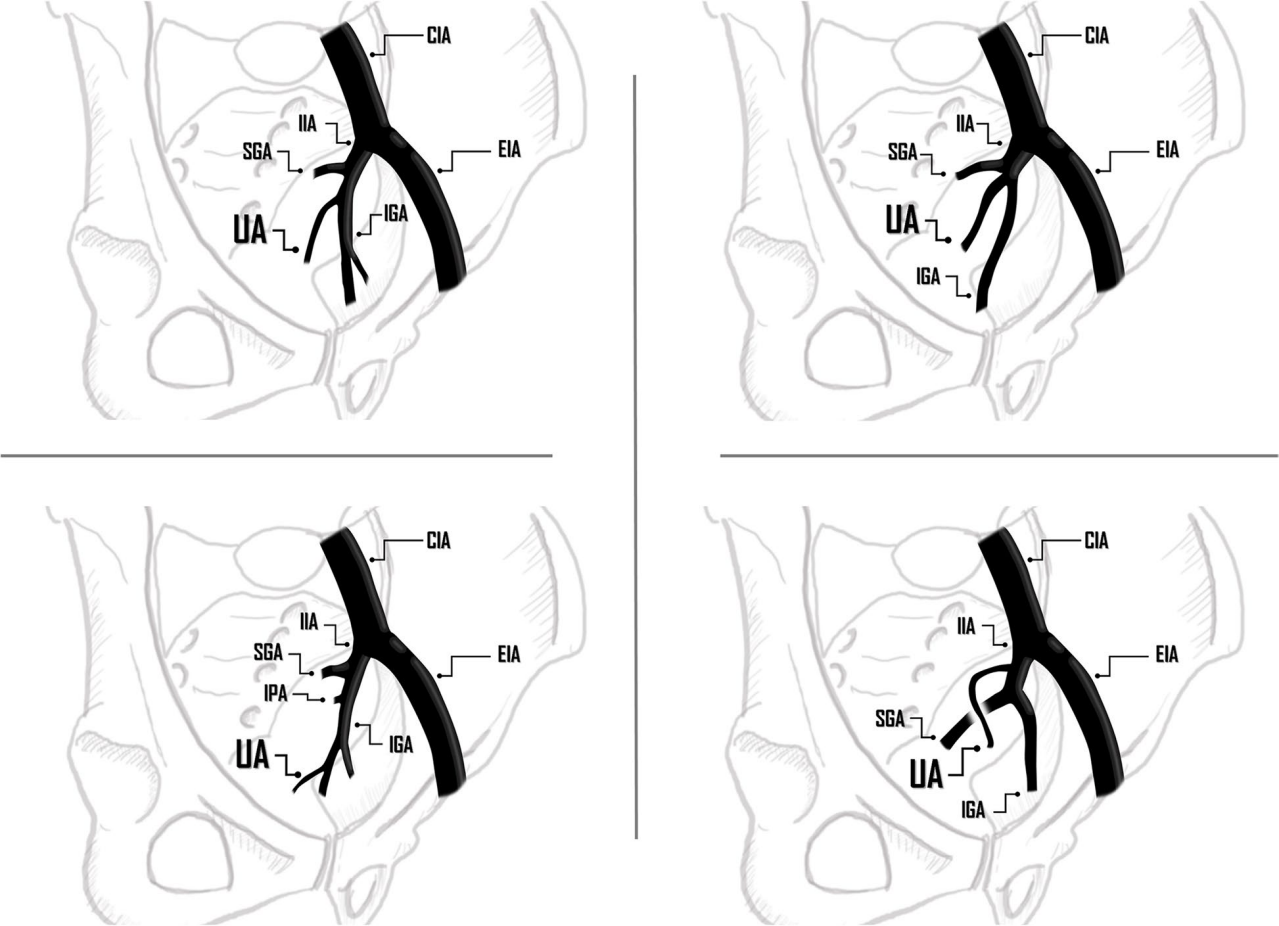
Schematic Drawing of anatomical variants at the origin of the uterine urtery, being the most common presentation its origin from the inferior gluteal artery or a common origin in the form of trifurcation along with the superior and inferior gluteal arteries. CIA: Common Iliac Artery, EIA: External Iliac Artery, IIA: Internal Iliac Artery, SGA: Superior Gluteal Artery, IGA: Inferior Gluteal Artery, UA: Uterine Artery, IPA: Internal Pudendal Artery

**Fig. 7:**
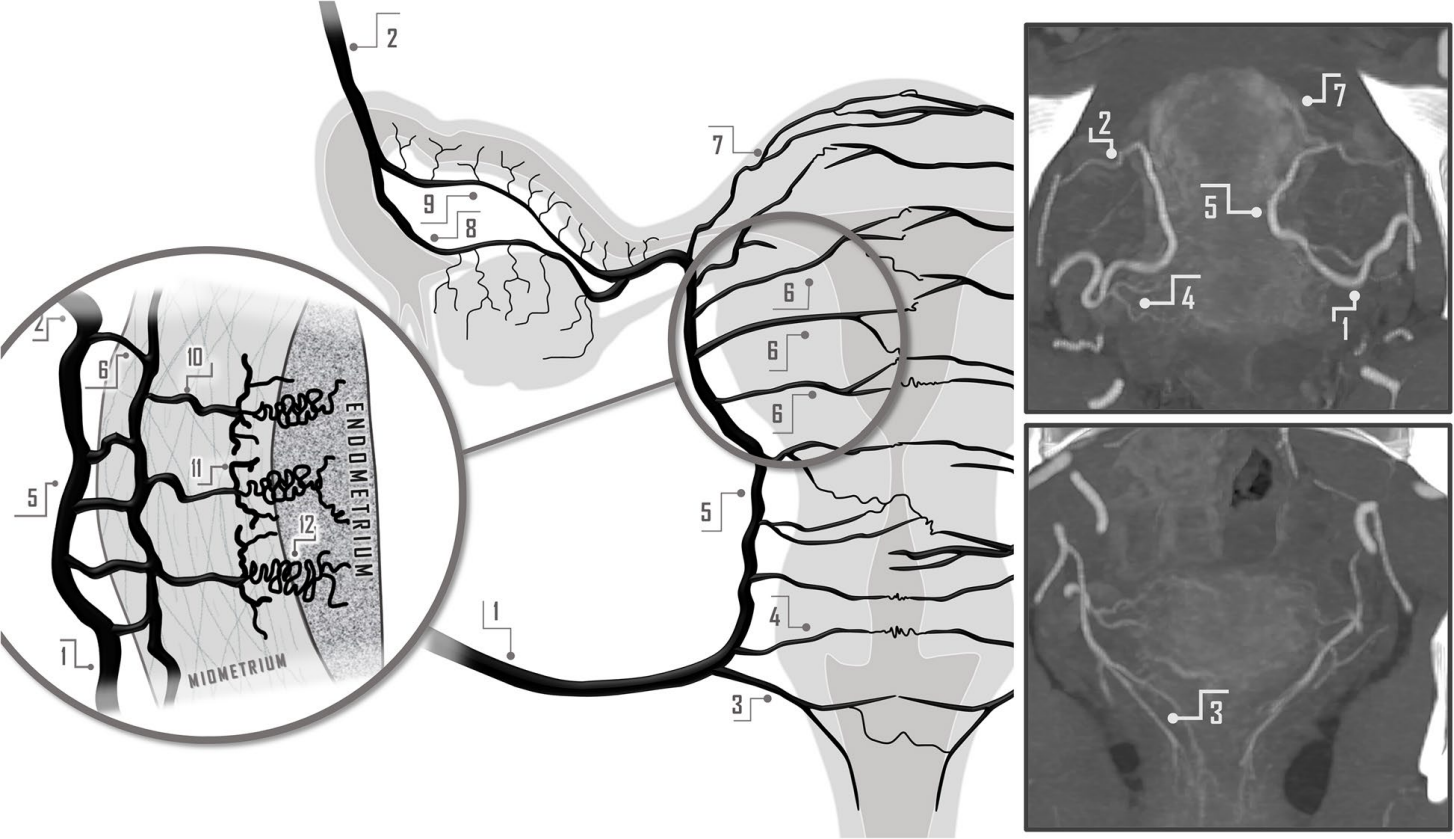
Arterial vascular supply to the uterus with angio-CT correlation

**Fig. 8:**
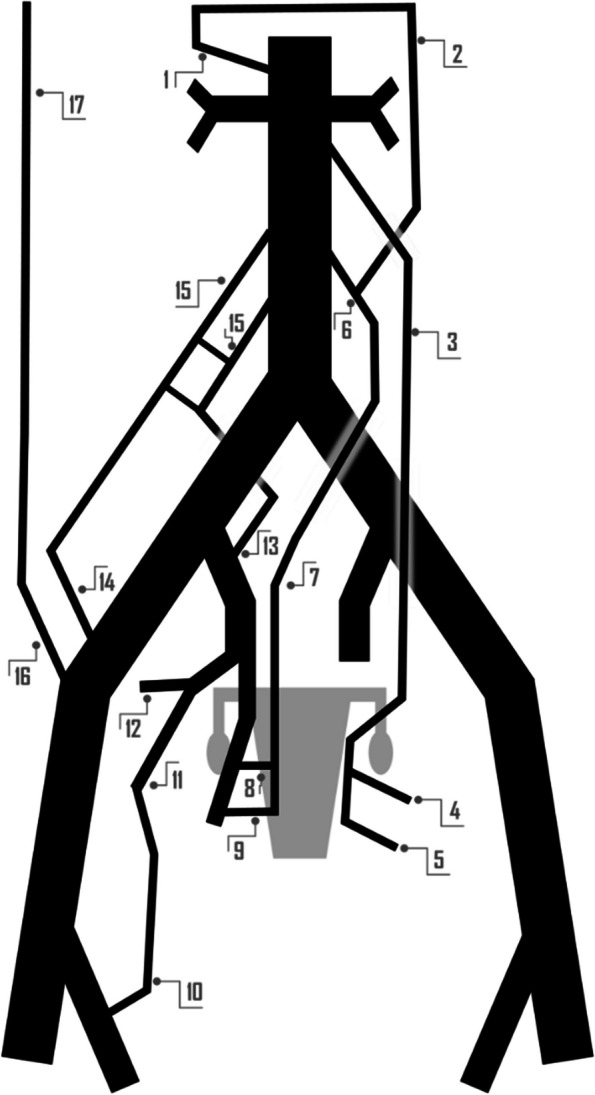
Collateral pathways to female pelvis vascular supply. 1) Superior mesenteric artery. 2) Riolan's arch (or marginal artery). 3) Ovarian artery. 4) Uterine artery. 5) Vaginal artery. 6) Inferior mesenteric artery. 7) Superior rectal artery. 8) Middle rectal artery. 9) Inferior rectal artery. 10) Femoral circumflex artery. 11) Inferior gluteal artery. 12) Superior gluteal artery. 13) Iliolumbar artery. 14) Deep circumflex iliac artery. 15) Lumbar artery. 16) Deep inferior epigastric artery. 17) Deep superior epigastric artery

Angiographically, active arterial bleeding manifests as contrast extravasation or the presence of a pseudoaneurysm. It is advisable to conduct a conclusive angiography with delayed acquisition images, enabling the identification of bleeding through collateral vessels [31]. The knowledge of normal female pelvis vascularization and collateral pathways has a special relevance in the context of recurrent PPH but also to prevent non-targeted embolization and to minimize the risk of vessel injury [20, 32]. The arterial vascularization of the female pelvis is illustrated in Figs. 6, 7 and 8.

Active bleeding in PPH is rarely seen [31]. When active bleeding site remains unidentified, empirical embolization procedure can be performed in PPH with a success rate over 95% by catheterizing and non-targeted deployment of embolic agent performed directly through a 4-F/5-F diagnostic catheter from the mid-terminal segment of the uterine artery. In this setting, bilateral embolization is recommended [20, 31]. Moreover, some authors have reported satisfactory and faster outcomes through non-selective embolization directly from the anterior division of the internal iliac artery [31, 33].

UAE in PPH is predominantly carried out employing non-permanent embolic materials such as resorbable gelatine sponge (GS) [20, 31]. GS expedite swift hemostasis by inducing mechanical obstruction and promoting thrombus formation within the vessel lumen which recanalizes in approximately 3 to 6 weeks [25]. The preparation of GS for embolization involved the creation of small torpedoes or a semi-liquid form from 1-2 mm pledgets (“slurry”). It is advisable to prepare this material simultaneously with the overall setup of the angio or Hybrid operating room, as it is a procedure that may consume valuable time. In this regard, having commercially prepared GS units pre-cut and pre-sized for delivery could streamline and expedite the procedure. The embolization endpoint is the attainment of complete stasis, as evidenced by a stagnant arteriogram [31]. According to Zhang et al., the clinical success rate of GS for PPH is 79 to 93.9%.

Calibrated microspheres have been used like permanent embolic agents for empirical non-selective UAE [25]. To prevent migration and uterine necrosis, particles larger than 700 μm [20] are recommended. The use of absorbable gelatin particles could provide an intermediate solution, acting as a temporary solution but also independent of the patient's clotting status in the context of PPH [34]. Coils or vascular plugs will be used for the embolization of collaterals to prevent unintended embolization.

Superselective embolization is performed when an identifiable bleeding point is observed, using GS pledgets, coils, or liquid embolic agents (ethylene–vinyl alcohol copolymer, EVOH, or N-butyl-2-cyanoacrylate, NBCA) [20, 31]. These liquid embolics have proven to be valuable in haemorrhage and AVMs superselective embolization procedures [35] and may be employed in cases of selective embolization in PPH. Superselective coaxial catheterization will be then required, avoiding vascular spasm, using 2.0-F or 2.4F microcatheters compatible with the embolic agent [8, 20, 31]. EVOH and NBCA are also useful in PPH cases where total quick vessel embolization is necessary [25] but also in cases when concerns arise about potential embolic agent migration [20, 36, 37]. Particularly in severe PPH, where consumptive coagulopathy can occur, these embolic agents become noteworthy to consider [5, 28].

### Uterus atony

UAE has emerged as the preferred treatment for PPH resulting from UA when conservative approach fails [3, 20]. UAE should be performed if the patient is haemodynamically stable enough to be moved to the angio suite [20]. In this context, consideration could be given to the placement of intrauterine tamponade balloon as the threshold for initiating a call to the Interventional Radiology Team, avoiding delays in the therapeutic algorithm [6].

**Fig. 9:**
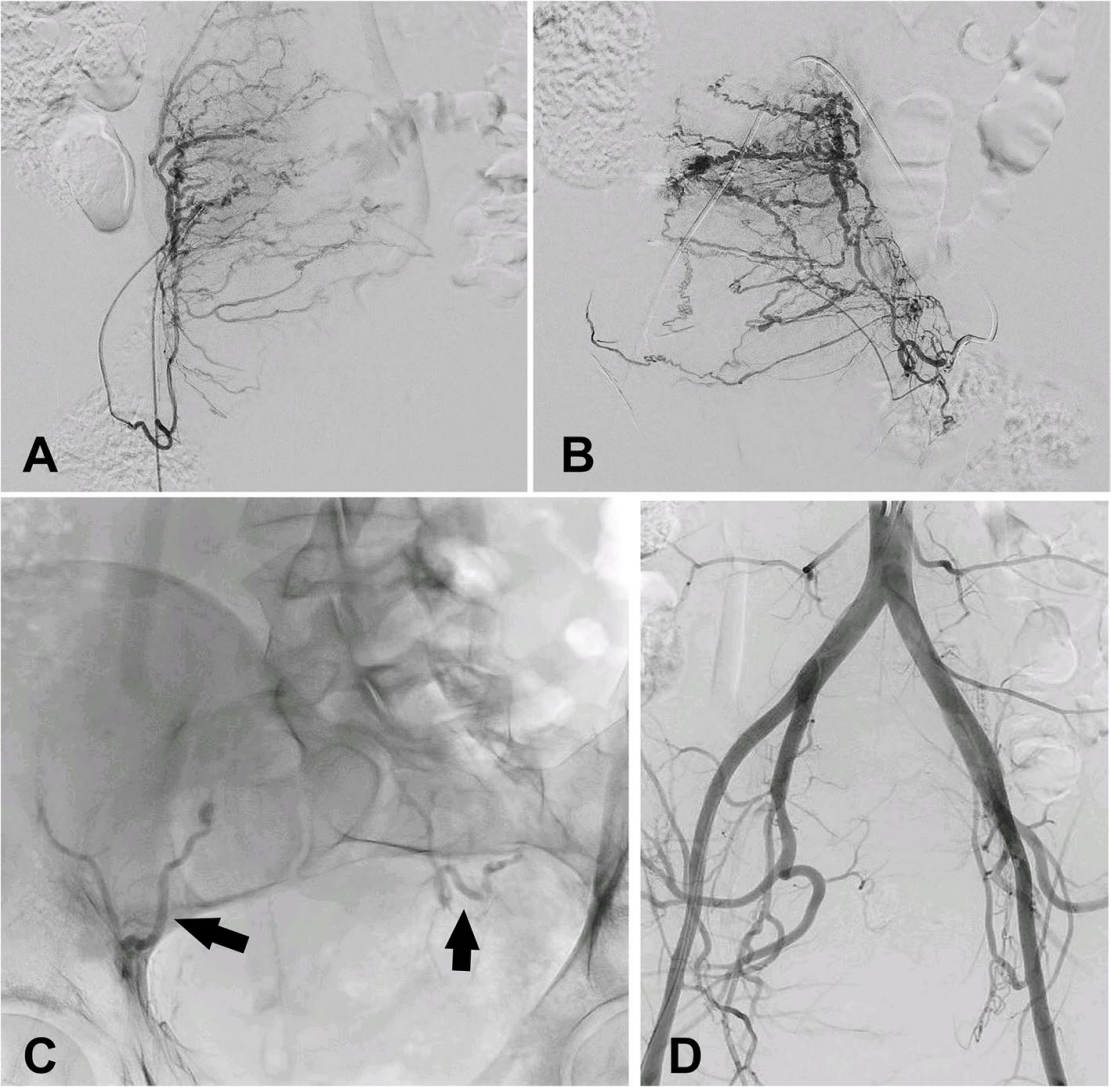
PPH in a 34-year-old woman due to uterine atony. selective catheterization of both uterine arteries via a right femoral approach (image A and B) with non-selective embolization using gelatin sponge (arrows in image B) demonstrating satisfactory outcomes in the follow-up angiography (image D)

In cases of PPH attributed to UA, the identification of active bleeding is detected only in 25 to 52% of cases, likely due to a diffuse, low-rate bleeding rather than a localized high-rate single-spot bleeding [31]. In cases of unidentified active bleeding, empirical embolization procedure can be performed as detailed above with GS (Fig. 9) and a success rate over 95% [31]. Microspheres over 700 µm can be also applied, however, PVA particles is not recommended in UA embolization, as they may cause uterine necrosis [31, 33]. In cases of active bleeding, the primary source often originates from the distal branches of the uterine or vaginal arteries and should be treated under superselective embolization as previously described [31].

### Trauma

The endovascular approach to uterine trauma lesions entails superselective catheterization of the bleeding site, as previously described, being effective as a treatment for pseudoaneurysms in uterine, vaginal, or cervical arteries, and according to Dohan et al., boasting a success rate exceeding 90% [8]. During angiography, a pseudoaneurysm appears as a localized accumulation of iodinated contrast material connected to the parent artery lumen, typically through a narrow neck (Fig. 2B, C) and embolization should be performed even if active bleeding is not evident [20]. Conversely to general principle in PPH embolization, in trauma-related cases or pseudoaneurysms, bilateral embolization might not be required in certain patients [8]. In case of massive bleeding, where the patient's coagulation integrity may be compromised, the use of EVOH or NBCA proves to be particularly useful in preventing rebleeding [35].

### Placenta accreta spectrum

The role of IR in the management of PAS has changed in the last decade [38–40], establishing itself today as a therapeutic approach that has demonstrated a significant decrease in intrapartum bleeding as well as the need for transfusions and hysterectomies [41, 42]. Amongst patients with PAS, blood loss during deliveries lacking IR procedures ranges from 3000 to 5000 mL, with transfusion required in up to 95% of cases. In deliveries assisted by IR techniques, the average blood loss varies from 586 to 1500 mL [43].

The most widely used IR technique for the management of PAS is the temporary occlusion of the internal iliac arteries (IIAs) through balloon inflation [44]. This procedure involves bilateral femoral artery approach and contralateral catheterization and placement of 6–7 mm balloon occlusion catheters, or Foley-type occlusion catheters, in the internal iliac artery or at its anterior bifurcation [44–46]. Then, the cesarean section is performed, and the balloons are inflated for hemostasia if needed. However, due to the extensive vascular collateralization and revascularization capacity of the pelvic territory [42] (Fig. 8) and according to the systematic review and meta-analysis by D ´Antonio et al. [39], this approach has achieved controversial clinical success rates between 50 and 80%, with complications rates up to 18% [39, 45], being the external iliac artery thrombosis the most serious complication as it necessitates urgent thrombectomy. Nevertheless, these results are influenced by heterogeneous sample results in terms of case numbers, degree of placental invasion, as well as varying inflation times [39, 44].

Pelvic hemostasis through aortic balloon inflation, including resuscitative endovascular balloon occlusion of the aorta (REBOA), are posited as an alternative to iliac occlusion in the context of PAS, aimed at diminishing the likelihood of revascularization through this network of pelvic vascular collaterals [42, 47]. The REBOA device is typically introduced through TFA, using a 7-F femoral sheath, although low-profile newer devices now allow for utilization through 4-F sheath [42, 48]. The final balloon placement is controlled via fluoroscopy and the temporary occlusion of the abdominal aorta is achieved by positioning and inflating the balloon in the distal aorta, ideally between the aortic bifurcation and the inferior mesenteric artery (zone 3). Following the extraction of the fetus, balloon is inflated for hemostasis if needed [42]. According to the study by Ioffe et al., the application of the REBOA catheter in the distal zone 3 of the aorta during cesarean hysterectomies for severe PAS disorders demonstrates a significant decrease in transfusions of ≥ 4 units compared to the control group without REBOA [49]. REBOA in PAS is also associated with adverse events linked to ischemia–reperfusion injury, and vessel damage [47]. According to Kluck et al., in comparison to IIAs occlusion, distal aortic occlusion results in up to a 46% reduction in blood loss, a 41.5% decrease in the number of transfusions, and a fivefold reduction in hysterectomy rates [42]. However, in line with the work of Mei et al., distal aortic occlusion, when compared to temporal iliac occlusion, does not appear to yield superior results in terms of blood loss associated with each technique in the context of PAS [50]. Given the novelty of this procedure in the obstetric population, there is limited literature on the use of this modality in cesarean hysterectomies performed for PAS disorders. However, based on the latest meta-analysis data, aortic occlusion outcomes appear to surpass other haemostatic management techniques in the context of PAS, including temporary iliac artery occlusion [51–54].

Nevertheless, given the lack of definitive control over hemostasis during childbirth using balloon-assisted arterial occlusion (either iliac or aortic), and where complications are not uncommon, it is imperative to take a step forward through intraprocedural UAE in the setting of PAS [13, 39–41, 46]. Consideration should be given to contemplate a proactive rather than reactive approach to intra-procedural haemorrhage in PAS with UAE.

**Fig. 10:**
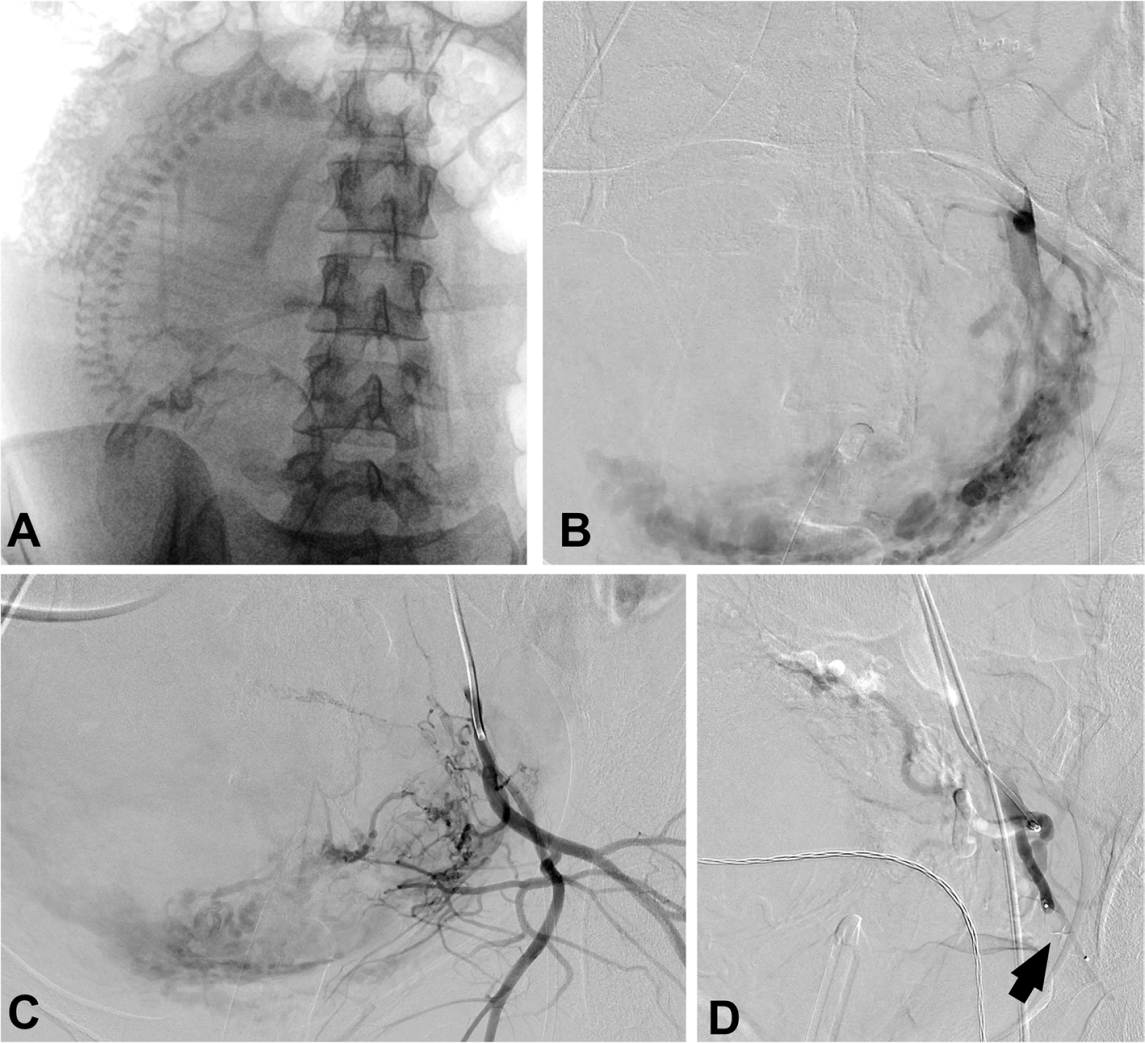
Uterine embolization in PAS procedure performed after cesarean histerectomy (image A-C) in a 41-year-old woman. Non-selective embolization was carried out using gelatine sponge as embolic agent. Vascular plug in UA is an easy and fast method for proximal embolization in colateral vessels (inferior glueteal artery, arrow in D) to prevent non-targeted embolization in this stressing scenario

The UAE procedure in cases of PAS closely parallels embolization performed in instances of UA (Fig. 10). According to Berman et al. [40], embolization in PAS after caesarean and prior to hysterectomy using 500-700 µm PVA particles, and compared to iliac occlusion, would significantly reduce both blood loss (713 ml vs. 2000 ml) and the need for blood transfusions (25% vs. 65% of transfusion requirement). However, although prophylactic UAE, using both particles or GS, instead of balloon arterial occlusion, has shown a significant reduction in intra-procedural haemorrhage [13, 40], according to the study by Chodraui-Filho et al. [43], simultaneous embolization alongside balloon-assisted arterial occlusion would not improve clinical outcomes compared to each procedure performed individually. However, it would be a point worth considering that the initial hemostatic control through the balloon occlusion would render the embolization procedure less stressful, safer, and with reduced emergency requirements and, even, avoiding unnecessary hysterectomies [41, 46].

Finally, the advantages of an interdisciplinary procedure in the management of PAS preferably, in a hybrid operating room, with the obstetrician performing the cesarean section first followed by prophylactic UAE, have been documented [13, 46]. This is an optimal environment with high-quality imaging, and time savings resulting from the elimination of patient transfer theatres.

### Retained products of conception

Invasive approaches in RPOC may result in massive bleeding during the procedures resulting in uncontrollable intraoperative bleeding. The endovascular management of PPH due to RPOC will be directed towards prophylactic embolization prior to the resection procedure to prevent a massive bleeding event [14, 18, 19] following diagnostic criteria previously detailed.

The technical approach to RPOC embolization prior to invasive management will be guided by the angiographic pattern [14, 16, 24] where RPOC have been described as: a) a sacular area with an angiographic pattern (AVM-like patter) highly reminiscent of a high-flow vascular malformation with a marked arteriovenous shunt [16, 24]; b) the presence of a diffuse area of contrast enhancement (blush) at the uterine level followed by an early venous return pattern, although without macroscopic evidence of direct venous drainage [14, 16]; c) uterine artery pseudoaneurysm [24]. Per Takaji et al., pelvic angiography revealed AVM-like patter or an aneurysm contiguous with dilated uterine arteries during the mid-arterial to capillary as the predominant angiographic observations [16]. Conversely, in the studies by Mathieu et al. and Kimura et al., the most prevalent RPOC finding in their respective series was identified as a 'blush' area pattern [24, 55].

**Fig. 11:**
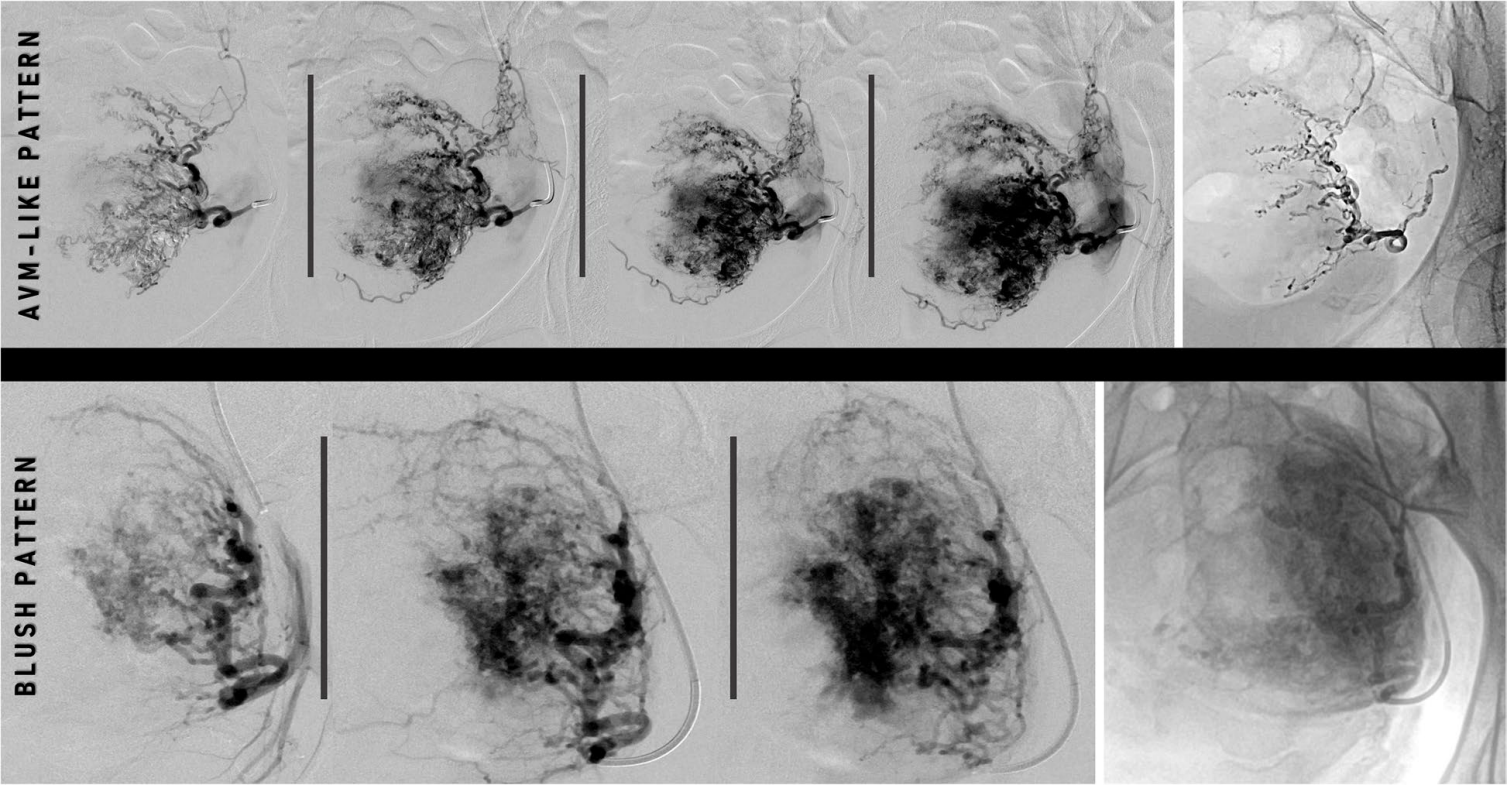
Top Line: 32-year-old woman with moderate secondary postpartum bleeding due to RPOC. Images sequence following contrast injection into the left uterine artery reveals an angiographic pattern of the lesion resembling the angiographic behavior of a vascular malformation, with drainage into the uterine and hypogastric veins during the capillary to venous phase (images 3–4 from left to right). Selective embolization of the lesion was performed using EVOH liquid agent (first from right, SquidPeri18LD®; Balt, Montmorency, France) with a satisfactory outcome. Bottom Line: 37-year-old woman with secondary severe PPH due to RPOC. Images sequence following contrast injection into the left uterine artery reveals an angiographic pattern of the lesion revealed as a poorly defined contrast enhancement area ('blush’ pattern) during the mid-arterial to capillary phase with no direct venous drainage. Same finding was observed on right side. UAE was performed using gelatin sponge as embolic agent in both left (first from right) and right uterine arteries with satisfactory result

The identification of an underlying lesion resembling an AVM will be achieved through superselective catheterization, followed by targeted embolization as if it were a genuine AVM. Conversely, when the angiographic pattern reveals a "blush" area, empirical non-selective embolization becomes the preferred approach [14, 16, 24] (Fig. 11).

Traditionally, GS has been employed in both slurry and torpedo forms, for both selective and non-selective UAE in the management of RPOC, with minimal post-UAE ischemic complications documented [16, 18, 20, 24, 26, 28]. According to Mathieu et al., this technique has reported clinical success rates of up to 100%, avoiding the need for D&C in over 95% of cases [24]. Microparticles, ranging from 700–1,200 μm, have also been also employed in non-selective UAE in RPOC. However, according to Bazeries et al., 25% of these patients required additional treatment suggesting that microparticles may be less effective than GS [14].

Selective embolization involves reaching the vascular nidus of the lesion or navigating as distally as possible under coaxial technique from the diagnostic catheter and the employment of a microcatheter as described above. EVOH and NBCA are well stablished embolic agents in the superselective approach of AVMs due to their ability to create a definitive cast that effectively infiltrates the nidus even in cases where direct access is challenging [56]. From that perspective, although they haven't been widely employed, both materials are considered a valid, safe, and effective alternative in the management of RPOC with AVM-like behaviour [36, 37].

## Conclusions

It is imperative to ensure a timely and accurate clinical and image-based diagnosis of PPH within an interdisciplinary approach that allows for the swift application of IR techniques from the outset. The non-selective UAE with GS is a rapid, effective, and safe technique in the management of PPH from UA, PAS, and RPOC with an angiographic "blush" pattern. Selective embolization will be performed when the bleeding point is identifiable or in RPOC with AVM-like angiographic pattern. It is necessary the conclusive incorporation of IR into clinical guidelines for PAS, along with the determination of the optimal action protocol across the diverse available techniques of temporary arterial occlusion, whether combined with UAE.

## Data Availability

Not applicable.

## Abbreviations

AVM: Arteriovenous Malformation
CT: Computed Tomography
D&C: Dilation and Curettage
DIC: Disseminated Intravascular Coagulation
EVOH: Ethylene–Vinyl Alcohol Copolymer
GS: Gelatine Sponge
IIA: Internal Iliac Arteries
MRI: Magnetic Resonance Imaging
NBCA: N-Butyl-2-Cyanoacrylate
PAS: Placenta Accreta Spectrum
PPH: Postpartum Haemorrhage
REBOA: Resuscitative Endovascular Balloon Occlusion of The Aorta
RPOC: Retained Products of Conception
TFA: Transfemoral Approach
TRA: Transradial Approach
UA: Uterine Atony
UAE: Uterine Artery Embolization
US: Ultrasound

## References

[1] Bienstock JL, Eke AC, Hueppchen NA (2021). Postpartum hemorrhage. Longo DL, editor. N Engl J Med.

[2] Mitta K, Tsakiridis I, Dagklis T, Grigoriadou R, Mamopoulos A, Athanasiadis A, et al. Incidence and risk factors for postpartum hemorrhage: a case-control study in a tertiary hospital in Greece. Medicina (Lithuania). 2023;59. 10.3390/medicina59061151 PMC10303199 37374355

[3] Escobar MF, Nassar AH, Theron G, Barnea ER, Nicholson W, Ramasauskaite D (2022). FIGO recommendations on the management of postpartum hemorrhage 2022. Int J Gynecol Obstet.

[4] Gallos I, Devall A, Martin J, Middleton L, Beeson L, Galadanci H (2023). Randomized trial of early detection and treatment of postpartum hemorrhage. N Engl J Med.

[5] Evensen A, Anderson JM (2017). Postpartum hemorrhage: prevention and treatment. Am Fam Physician.

[6] Weeks AD, Akinola OI, Amorim M, Carvalho B, Deneux-Tharaux C, Liabsuetrakul T (2022). World health organization recommendation for using uterine balloon tamponade to treat postpartum hemorrhage. Obstet Gynecol.

[7] Sebghati M, Chandraharan E (2017). An update on the risk factors for and management of obstetric haemorrhage. Women’s Health.

[8] Dohan A, Soyer P, Subhani A, Hequet D, Fargeaudou Y, Morel O (2013). Postpartum hemorrhage resulting from pelvic pseudoaneurysm: a retrospective analysis of 588 consecutive cases treated by arterial embolization. Cardiovasc Intervent Radiol.

[9] Lee NK, Kim S, Lee JW, Sol YL, Kim CW, Hyun Sung K (2010). Postpartum hemorrhage: clinical and radiologic aspects. Eur J Radiol.

[10] Morlando M, Collins S (2020). Placenta accreta spectrum disorders: challenges, risks, and management strategies. Int J Womens Health.

[11] Silver RM, Branch DW (2018). Placenta accreta spectrum. N Engl J Med.

[12] Cali G, Forlani F, Giambanco L, Amico ML, Vallone M, Puccio G (2014). Prophylactic use of intravascular balloon catheters in women with placenta accreta, increta and percreta. Eur J Obstet Gynecol Reprod Biol.

[13] Melber DJ, Berman ZT, Jacobs MB, Picel AC, Conturie CL, Zhang-Rutledge K (2021). Placenta accreta spectrum treatment with intraoperative multivessel embolization: the PASTIME protocol. Am J Obstet Gynecol.

[14] Bazeries P, Paisant-Thouveny F, Yahya S, Bouvier A, Nedelcu C, Boussion F (2017). Uterine artery embolization for retained products of conception with marked vascularity: a safe and efficient first-line treatment. Cardiovasc Intervent Radiol.

[15] Groszmann YS, Healy Murphy AL, Benacerraf BR (2018). Diagnosis and management of patients with enhanced myometrial vascularity associated with retained products of conception. Ultrasound Obstet Gynecol.

[16] Takaji R, Kiyosue H, Maruno M, Hongo N, Shimada R, Ide S (2021). Angiographic features and transarterial embolization of retained placenta with abnormal vaginal bleeding. CVIR Endovasc.

[17] Foreste V, Gallo A, Manzi A, Riccardi C, Carugno J, Sardo A (2021). Hysteroscopy and retained products of conception: an update. Gynecol Minim Invasive Ther.

[18] Kitahara T, Sato Y, Kakui K, Tatsumi K, Fujiwara H, Konishi I (2011). Management of retained products of conception with marked vascularity. J Obstet Gynaecol Res.

[19] Schulte RL, Fox R, Anderson J, Young N, Davis L, Saxton V (2023). Medical management of retained products of conception: a prospective observational study. Eur J Obstet Gynecol Reprod Biol.

[20] Rand T, Patel R, Magerle W, Uberoi R (2020). CIRSE standards of practice on gynaecological and obstetric haemorrhage. CVIR Endovasc.

[21] Collins SL, Ashcroft A, Braun T, Calda P, Langhoff-Roos J, Morel O (2016). Proposal for standardized ultrasound descriptors of abnormally invasive placenta (AIP). Ultrasound Obstet Gynecol.

[22] Morel O, Collins SL, Uzan-Augui J, Masselli G, Duan J, Chabot-Lecoanet AC (2019). A proposal for standardized magnetic resonance imaging (MRI) descriptors of abnormally invasive placenta (AIP) – From the International Society for AIP. Diagn Interv Imaging.

[23] Vyas S, Choi HH, Whetstone S, Priyanka J, Liina P, Shum DJ (2021). Ultrasound features help identify patients who can undergo noninvasive management for suspected retained products of conception: a single institutional experience. Abdominal Radiol.

[24] Mathieu E, Riethmuller D, Delouche A, Sicot M, Teyssier Y, Finas M (2022). Management of symptomatic vascularized retained products of conception by proximal uterine artery embolization with gelatin sponge torpedoes. J Vasc Interv Radiol.

[25] Lindquist JD, Vogelzang RL (2018). Pelvic artery embolization for treatment of postpartum hemorrhage. Semin Intervent Radiol.

[26] Matsuzaki S, Lee M, Nagase Y, Jitsumori M, Matsuzaki S, Maeda M (2021). A systematic review and meta-analysis of obstetric and maternal outcomes after prior uterine artery embolization. Sci Rep.

[27] Radan AP, Schneider S, Zdanowicz JA, Raio L, Mertineit N, Heverhagen JT (2022). Obstetrical and fertility outcomes following transcatheter pelvic arterial embolization for postpartum hemorrhage: a cohort follow-up study. Life.

[28] Kimura Y, Osuga K, Nagai K, Hongyo H, Tanaka K, Ono Y (2020). The efficacy of uterine artery embolization with gelatin sponge for retained products of conception with bleeding and future pregnancy outcomes. CVIR Endovasc.

[29] Himiniuc LM, Murarasu M, Toma B, Popovici R, Grigore A-M, Scripcariu I-S (2021). medicina transradial embolization, an underused type of uterine artery embolization approach: a systematic review. Medicina (B Aires).

[30] Khayrutdinov E, Vorontsov I, Arablinskiy A, Shcherbakov D, Gromov D (2021). A randomized comparison of transradial and transfemoral access in uterine artery embolization. Diagn Interv Radiol.

[31] Soyer P, Dohan A, Dautry R, Guerrache Y, Ricbourg A, Gayat E (2015). Transcatheter arterial embolization for postpartum hemorrhage: indications, technique, results, and complications. Cardiovasc Intervent Radiol.

[32] Gomez-Jorge J, Keyoung A, Levy EB, Spies JB (2003). Uterine artery anatomy relevant to uterine leiomyomata embolization. Cardiovasc Intervent Radiol.

[33] Zhang XQ, Chen XT, Zhang YT, Mai CX (2021). The Emergent pelvic artery embolization in the management of postpartum hemorrhage: a systematic review and metaanalysis. Obstet Gynecol Surv.

[34] Maclean D, Vigneswaran G, Bryant T, Modi S, Hacking N (2021). A retrospective cohort study comparing a novel, spherical, resorbable particle against five established embolic agents for uterine fibroid embolisation. Clin Radiol.

[35] Piacentino F, Fontana F, Curti M, Macchi E, Coppola A, Ossola C (2021). Non-adhesive liquid embolic agents in extra-cranial district: state of the art and review of the literature. J Clin Med.

[36] Barral P-A, Saeed-Kilani M, Tradi F, Dabadie A, Izaaryene J, Soussan J (2017). Transcatheter arterial embolization with ethylene vinyl alcohol copolymer (Onyx) for the treatment of hemorrhage due to uterine arteriovenous malformations. Diagn Interv Imaging.

[37] Hamaguchi S, Okura N, Yoshimatsu M, Ogawa Y, Takizawa K, Nakajima Y (2012). A case of retained placenta increta successfully treated via uterine arterial embolization using N-Butyl 2-Cyanoacrylate. J Minim Invasive Gynecol.

[38] Heidemann B (2011). Interventional radiology in the treatment of morbidly adherent placenta: are we asking the right questions?. Int J Obstet Anesth.

[39] D’Antonio F, Iacovelli A, Liberati M, Leombroni M, Murgano D, Cali G (2019). Role of interventional radiology in pregnancy complicated by placenta accreta spectrum disorder: systematic review and meta-analysis. Ultrasound Obstet Gynecol.

[40] Berman ZT, Boone CE, Melber DJ, Ballas J, Parikh R, Ramos G (2023). Intraoperative multivessel embolization reduces blood loss and transfusion requirements compared to internal iliac artery balloon placement during cesarean hysterectomy for placenta accreta spectrum. J Vascular Interv Radiol.

[41] Alam B, Nasir F, Akbari AR, Alali B, Khalil Z (2023). A Review and comparison of the efficacy of prophylactic interventional radiological arterial occlusions in placenta accreta spectrum patients: a meta-analysis. Acad Radiol.

[42] Kluck SL, Russo RM, Appel NB, Frankfurt AI, Weltge C, Shimer T (2023). Aortic balloon occlusion in distal zone 3 reduces blood loss from obstetric hemorrhage in placenta accreta spectrum. J Trauma Acute Care Surg.

[43] Filho SFC, Monsignore LM, Freitas RK, Nakiri GS, Cavalli RDC, Duarte G (2019). Can the combination of internal iliac temporary occlusion and uterine artery embolization reduce bleeding and the need for intraoperative blood transfusion in cases of invasive placentation?. Clinics (Sao Paulo).

[44] Picel AC, Wolford B, Cochran RL, Ramos GA, Roberts AC (2018). Prophylactic internal iliac artery occlusion balloon placement to reduce operative blood loss in patients with invasive placenta. J Vasc Interv Radiol.

[45] Salim R, Chulski A, Romano S, Garmi G, Rudin M, Shalev E (2015). Precesarean prophylactic balloon catheters for suspected placenta accreta a randomized controlled trial. Obstet Gynecol.

[46] Sebastian B, Rajesh U, Scott PM, Sayeed S, Robinson GJ, Ettles DF (2023). Prophylactic uterine artery embolization in placenta accreta spectrum—An active intervention to reduce morbidity and promote uterine preservation. J Vasc Interv Radiol.

[47] Nieto-Calvache AJ, Hidalgo-Cardona A, Lopez-Girón MC, Rodriguez F, Ordoñez C, Garcia AF (2022). Arterial thrombosis after REBOA use in placenta accreta spectrum: a case series. J Matern Fetal Neonatal Med.

[48] Power A, Parekh A, Scallan O, Smith S, Novick T, Parry N, et al. Size matters: First-in-human study of a novel 4 French REBOA device. Trauma Surg Acute Care Open. 2021;6. 10.1136/tsaco-2020-000617 PMC7798668 33490605

[49] Ioffe YJM, Burruss S, Yao R, Tse B, Cryer A, Mukherjee K (2021). When the balloon goes up, blood transfusion goes down: a pilot study of REBOA in placenta accreta spectrum disorders. Trauma Surg Acute Care Open.

[50] Mei Y, Zhao H, Zhou H, Jing H, Lin Y (2019). Comparison of infrarenal aortic balloon occlusion with internal iliac artery balloon occlusion for patients with placenta accreta. BMC Pregnancy Childbirth.

[51] Theodorou CM, Rinderknecht TN, Girda E, Galante JM, Russo RM (2022). Fetal and neonatal outcomes following maternal aortic balloon occlusion for hemorrhage in pregnancy: a review of the literature. J Trauma Acute Care Surg.

[52] Chen L, Wang X, Wang H, Li Q, Shan N, Qi H (2019). Clinical evaluation of prophylactic abdominal aortic balloon occlusion in patients with placenta accreta: a systematic review and meta-analysis. BMC Pregnancy Childbirth.

[53] Ordoñez CA, Manzano-Nunez R, Parra MW, Rasmussen TE, Nieto AJ, Herrera-Escobar JP (2018). Prophylactic use of resuscitative endovascular balloon occlusion of the aorta in women with abnormal placentation: a systematic review, meta-analysis, and case series. J Trauma Acute Care Surg.

[54] Li K, Zou Y, Sun J, Wen H (2018). Prophylactic balloon occlusion of internal iliac arteries, common iliac arteries and infrarenal abdominal aorta in pregnancies complicated by placenta accreta: a retrospective cohort study. Eur Radiol.

[55] Kimura Y, Osuga K, Nagai K, Hongyo H, Tanaka K, Ono Y (2020). The efficacy of uterine artery embolization with gelatin sponge for retained products of conception with bleeding and future pregnancy outcomes. CVIR Endovasc.

[56] Vollherbst DF, Chapot R, Bendszus M, Möhlenbruch MA (2022). Glue, Onyx, Squid or PHIL? Liquid embolic agents for the embolization of cerebral arteriovenous malformations and dural arteriovenous fistulas. Clin Neuroradiol.

